# The Graft-Versus-Leukemia Effect in AML

**DOI:** 10.3389/fonc.2019.01217

**Published:** 2019-11-19

**Authors:** Connor Sweeney, Paresh Vyas

**Affiliations:** ^1^MRC Molecular Haematology Unit, Oxford Biomedical Research Centre, MRC Weatherall Institute of Molecular Medicine, University of Oxford, Oxford, United Kingdom; ^2^Department of Haematology, Oxford University Hospitals NHS Foundation Trust, Oxford, United Kingdom

**Keywords:** acute myeloid leukemia, stem cell transplantation, graft-versus-leukemia, graft-versus-host disease, T cells, antigens

## Abstract

Allogeneic hematopoietic stem cell transplantation (allo-SCT) is the most established and commonly used cellular immunotherapy in cancer care. It is the most potent anti-leukemic therapy in patients with acute myeloid leukemia (AML) and is routinely used with curative intent in patients with intermediate and poor risk disease. Donor T cells, and possibly other immune cells, eliminate residual leukemia cells after prior (radio)chemotherapy. This immune-mediated response is known as graft-versus-leukemia (GvL). Donor alloimmune responses can also be directed against healthy tissues, which is known as graft-versus-host disease (GvHD). GvHD and GvL often co-occur and, therefore, a major barrier to exploiting the full immunotherapeutic benefit of donor immune cells against patient leukemia is the immunosuppression required to treat GvHD. However, curative responses to allo-SCT and GvHD do not always occur together, suggesting that these two immune responses could be de-coupled in some patients. To make further progress in successfully promoting GvL without GvHD, we must transform our limited understanding of the cellular and molecular basis of GvL and GvHD. Specifically, in most patients we do not understand the antigenic basis of immune responses in GvL and GvHD. Identification of antigens important for GvL but not GvHD, and vice versa, could impact on donor selection, allow us to track GvL immune responses and begin to specifically harness and strengthen anti-leukemic immune responses against patient AML cells, whilst minimizing the toxicity of GvHD.

## Introduction

Acute myeloid leukemia (AML) is the commonest aggressive leukemia in adults. It is the most frequent indication for allo-SCT, accounting for 36% of transplants in Europe (1). Medically fit patients are treated with cytotoxic chemotherapy and are stratified according to their risk of relapse, which is based on genetic features of their leukemia and response to initial treatment. Approximately 70–80% of patients aged under 60, and 50% of older patients, achieve a remission with induction chemotherapy (2, 3). Despite achieving a remission, without further treatment most patients would subsequently relapse, usually within 6 months, and relapse is associated with poor prognosis. Therefore, post-remission therapy aims to reduce relapse by eliminating residual leukemia cells (4).

Patients at higher risk of relapse receive an allo-SCT, which remains the most effective anti-leukemic curative treatment for the majority of AML patients with intermediate and poor risk disease (5). Prior to transplantation a patient receives conditioning, which consists of high doses of chemotherapy, with or without total body irradiation (TBI). Conditioning aims to kill cancerous cells but also reduces native bone marrow hematopoietic and immune cells. Subsequent transplantation of hematopoietic stem/progenitor and immune cells from a healthy donor leads to reconstitution of normal hematopoietic and immune cells. Crucially, alloreactivity of donor T cells against the patient's leukemia is responsible for the graft-versus-leukemia (GvL) effect, which is a major mechanism for the curative effect of allo-SCT (6). However, this must be balanced against alloreactivity against normal tissues, which manifests as graft-versus-host disease (GvHD), a multi-system disorder that initially commonly affects the skin, gastrointestinal tract, liver, and lungs but later can affect almost any organ (7, 8).

Strategies to ameliorate the negative immunological effects include T cell depletion of grafts and treating recipients with immunosuppressive drugs (9). However, these can also dampen the desired anti-tumor (GvL) responses, increasing the risk of relapse, and therefore a careful balance is required. After recovering from the toxicity of conditioning, if there is a concern about lack of a GvL response, for example due to loss of donor chimerism or evidence of molecular relapse, infusions of donor T cells can be administered as donor lymphocyte infusions (DLIs) in an attempt to produce a GvL response, with an associated risk of inducing GvHD (10, 11).

The overall survival (OS) of AML patients treated by allo-SCT remains modest at ~50% at 3 years, which is largely due to relapse and treatment-related mortality (GvHD and infection), emphasizing the need to improve upon current treatment approaches (12).

## Historical Perspective

Allo-SCT has been administered for over 50 years and remains the commonest and most effective cellular immunotherapy for myeloid malignancy. When first introduced, the aim of transplantation was to permit the delivery of high doses of chemotherapy that would otherwise be limited by toxicity to the native hematopoietic system. The first suggestion of a donor immune response against leukemia came in 1956 following mouse transplantation experiments, where murine leukemia relapses appeared to be reduced following transplantation with allogeneic bone marrow (from a different mouse strain) compared with the syngeneic marrow (same mouse strain) (13). Some of the animals treated with allogeneic transplantation died from a “wasting syndrome” with diarrhea that likely represented GvHD.

The first human allogeneic bone marrow transplantations were reported the following year by Thomas et al. (14). Six patients were treated with conditioning chemotherapy and radiotherapy, followed by an infusion of bone marrow from a healthy donor. Only two of the six patients showed evidence of engraftment and all died within 100 days of transplantation. Donors and patients were not matched for histocompatibility, as little was known about human histocompatibility antigens at this time.

The identification of human leukocyte antigens (HLA) and the development of methods to type these antigens enabled transplant programs to improve outcomes by patient-donor histocompatibility matching (15). The HLA locus is on the short arm of chromosome 6 and is one of the most polymorphic regions of the human genome. The polymorphic genetic diversity is an adaptive feature to facilitate presentation of the large repertoire of microbial antigens and is under host-pathogen co-evolution (16). The first HLA antigen (HLA-A2) was discovered in 1958 by Jean Dausset, who studied the sera from patients who had received multiple blood transfusions (17). He found that sera from some of these patients agglutinated leukocytes from 11 of 19 individuals tested, but not leukocytes from the donor of sera, suggesting the presence of an alloantigen (18). Further HLA antigens were characterized in subsequent years by Thorsby (18), van Rood et al. (19), and Payne et al. (20). The clinical importance of HLA matching in kidney transplantation was realized during the 1960s. Grafts between unrelated individuals are associated with a high likelihood of major incompatibility due to the polymorphic nature of the HLA locus. However, within a family there are only 4 haplotypes (2 from each parent). In 1965, Terasaki and colleagues reported improved kidney graft survival from HLA-matched donors and by the early 1970s it was evident that kidneys transplanted between HLA-identical siblings was the optimal combination (18).

In 1965, Mathé reported histocompatibility testing of bone marrow donors for a patient with acute lymphoblastic leukemia (ALL) (21). The patient was given grafts from six family members and developed a “secondary syndrome,” later to be described as GvHD, involving the skin, gastrointestinal tract, and liver. Six months after the transplant, further bone marrow was administered with the aim of enhancing the immune response against leukemia. In order to choose the donor least likely to reactivate a secondary syndrome, histocompatibility testing was performed by giving the patient skin grafts from each donor. The donor was selected whose skin graft was not rejected, but this still led to a steroid-responsive “secondary syndrome” (22).

The development of techniques to type HLA in patients and donors in the mid/late 1960s enabled E. Donnall Thomas to open an allogeneic bone marrow transplantation program in Seattle using HLA-matched donors for patients with acute leukemia. In 1977, they reported 100 transplantations for relapsed and refractory acute leukemia, using chemotherapy and radiation therapy conditioning (23). HLA matching was performed using mixed leukocyte culture and all transplants used HLA-identical related donors. Although only 13 patients were disease free after 1–4.5 years follow-up in this case series, administering transplantation earlier in the course of AML resulted in a cure rate of 50% in patients transplanted in first remission (24). Crucially, Thomas appreciated that the donor immune system likely plays a key role in eliminating residual leukemia cells. Although survival was reduced in patients with severe GvHD, most patients did not die of relapse. Furthermore, patients with GvHD relapsed later than those without GvHD (23, 25). In 1990, E. Donnall Thomas won a Nobel Prize for his discoveries concerning cell transplantation in the treatment of human disease.

## Donor Selection

Despite being the preferred donor, a HLA-matched sibling is available for only 30% of patients. The use of HLA-matched unrelated donors for stem cell transplantation drastically increased the number of patients who could be treated (26). Although the HLA locus is highly polymorphic, haplotypes are conserved in populations due to linkage disequilibrium. In the years after the first unrelated donor transplant, donor registries were established, but due to large variation in ethnic representation, the chance of finding a full HLA match varied widely according to ethnicity (27). Therefore, international collaboration has been essential for the establishment of a global donor registry. The International Bone Marrow Transplant Registry (IBMTR) was founded in 1972 to record transplant outcome data. In 1974, the European Group for Blood and Marrow Transplantation (EBMT) was formed and in 1988 Bone Marrow Donors Worldwide was established. There are currently over 34 million registered donors worldwide and almost 800,000 cord blood units (28). Although a donor will be found for most patients, a high resolution HLA-matched donor will not be available for all, in particular for patients that are not of white European descent, as the ethnic diversity of patients is not reflected in donor registries (29).

In the absence of a HLA-matched donor, the use of alternative donor allo-SCTs has increased the pool of available donors. This involves either a HLA-mismatched unrelated donor, haploidentical related donor or an umbilical cord blood transplant. Mismatched allo-SCTs generally have a mismatch at 1 or 2 HLA alleles (HLA-A, -B, -C, -DRB1, or -DQB1), as a greater degree of mismatch is associated with unacceptably high risks of GvHD and non-relapse mortality (15).

Haploidentical related donor transplants are from siblings, parents or children of the patient who share at least 50% of HLA alleles. As most patients have at least one haploidentical first-degree relative, this approach has enabled a donor to be found for almost all patients, which is particularly important for ethnic minority groups that are under-represented in donor registries. Due to bidirectional alloreactive T cell responses, the first haploidentical transplants were associated with high mortality from GvHD and also a host-versus-graft reaction that resulted in rejection of transplanted cells (30). Improvement in T cell depletion methods have reduced GvHD and graft failure following haploidentical allo-SCT. In 2005, a phase II study of 101 patients treated with haploidentical allo-SCT used positive selection for CD34^+^ cells to T cell deplete grafts and administered high doses of CD34^+^ stem cells to recipients. They reported engraftment in 93% of patients, acute GvHD in 8% and chronic GvHD in 7% of evaluable patients. However, 36.5% of patient died of non-relapse causes, mainly due to infection (31). This highlights a major challenge of administering haploidentical allo-SCT. Although intensive conditioning of the recipient and T cell depletion of the graft improves GvHD and hematopoietic engraftment, immune reconstitution is poor, leading to opportunistic infections. Nevertheless, outcomes following haploidentical allo-SCT have improved with refinements in T cell depletion, including the use of cyclophosphamide in a narrow time window post-transplantation (32). The use of haploidentical transplants has been increasing in recent years and EBMT reported that 14.7% of allo-SCTs in 2017 used haploidentical donors (33).

Umbilical cord blood transplantation is an alternative source of hematopoietic stem cells for transplantation. Naivety of immune cells in cord blood permits less stringent HLA matching. However, due to the relatively low number of stem cells in a unit, adult transplants require either two cord units or *ex vivo* expansion of stem cells. Furthermore, the naivety of immune cells leads to an increase in opportunistic infections. As the use of haploidentical donors has increased, cord blood transplants have reduced and 2% of allo-SCTs reported by EBMT in 2017 used cord blood donations (33).

## Allogeneic Stem Cell Transplantation for AML

Although allo-SCT reduces relapse, non-relapse mortality due to complications of the transplant including GvHD and infection will counterbalance this beneficial effect in many patients. Therefore, when deciding which individuals will benefit from allo-SCT, there must be a patient-specific evaluation. The European LeukemiaNet (ELN) AML Working Party proposes a dynamic risk assessment that integrates the cytogenetic and molecular genetic features of AML at diagnosis with the patient's response to induction therapy to estimate the risk of relapse after consolidation treatment with either allo-SCT or chemotherapy. This relapse risk is balanced against the non-relapse mortality from allo-SCT that is estimated using the patient's co-morbidities with the hematopoietic cell transplantation comorbidity index, HCT-CI (34) (Table 1). The ELN suggest that if, based on an individual's risk assessment, the disease-free survival is predicted to improve by at least 10%, allo-SCT should be recommended. In the absence of significant co-morbidities, this translates to intermediate and poor risk patients.

**Table 1:** European LeukemiaNet (ELN) recommendations for allogeneic stem cell transplantation in patients with AML in first complete remission.

**AML risk group**	**Risk assessment**	**Risk of relapse following consolidation treatment**		**Non-relapse mortality risk that would indicate allo-SCT as consolidation treatment**	
		Chemotherapy^*^ (%)	**Allo-SCT (%)**	**HCT-CI score**	**Non-relapse mortality (%)**
Good	*t*(8; 21) with WBC ≤20 Inv(16)/*t*(16; 16) Mutated *CEBPA* (bi-allelic) Mutated *NPM1* (No *FLT3–*ITD mutation) Early first complete remission (after first cycle of chemotherapy) and MRD negative	35–40	15–20	0	10–15
Intermediate	*t*(8; 21) with WBC >20 Cytogenetically normal (or loss of X and Y chromosomes), WBC count ≤100 and early first complete remission	50–55	20–25	≤2	<20–25
Poor	Otherwise good or intermediate, but not in complete remission after first cycle of chemotherapy Normal cytogenetics and WBC >100 Abnormal cytogenetics	70–80	30–40	≤3–4	<30
Very poor	Monosomal karyotype Abn3q26 Enhanced Evi-1 expression	>90	40-50	≤5	<40

ELN 2012 patient-specific risk assessment of AML relapse and non-relapse mortality following allo-SCT compared with chemotherapy consolidation. Recommendation of allo-SCT if the individual patient's disease-free survival benefit is at least 10%.

* *Chemotherapy consolidation includes option of high-dose chemotherapy and autologous stem cell rescue. AML, acute myeloid leukemia; allo-SCT, allogeneic stem cell transplantation; HCT-CI, hematopoietic cell transplantation comorbidity index; CEBPA, CCAAT/enhancer-binding protein α; NPM1, nucleophosmin; FLT3-ITD, fms-like tyrosine kinase receptor-3 internal tandem duplication; monosomal karyotype, defined by presence of either two or more autosomal monosomies or one monosomy plus one or more structural aberrations; Evi-1, Ecotropic viral integration site 1; WBC, white blood cell count at diagnosis; MRD, minimal residual disease. Adapted from Cornelissen et al. (35)*.

The ELN genetic risk stratification has been refined in recent years, reflecting a deeper understanding of the genomic landscape of AML, and mutations in genes such as *RUNX1, ASXL1*, and *TP53* now contribute to the adverse risk category (36, 37). Assessment of post-treatment minimal residual disease (MRD) provides additional prognostic information that complements pre-treatment genetic risk stratification. The presence of low amounts of MRD has been consistently associated with increased relapse and reduced OS in AML (38). Two approaches may be used for MRD detection: (1) multiparameter flow cytometry, and (2) molecular techniques, including real-time quantitative PCR (RT-qPCR) and next generation sequencing (NGS). MRD using flow cytometry commonly involves the identification of a leukemia-associated immunophenotype for the individual patient that differs from normal hematopoietic cells (39). RT-qPCR assays are available for MRD detection of specific genetic lesions found in sub-groups of patients with AML, including *NPM1* mutations, *CBFB-MYH11, RUNX1-RUNX1T1*, and *BCR-ABL1* fusion genes. As a molecular marker can be detected in the majority of cases, NGS offers the possibility of tracking additional molecular markers in the future. However, validation of markers is still required, as mutations in genes associated with pre-leukemic clones (e.g., *DNMT3A, TET2*, and *ASXL1*) are frequently detected at remission but are poorly predictive of relapse (40). A key goal is to be able to use MRD data to identify early recurrence of leukemia and guide post-remission therapy. This could offer the opportunity to intervene pre-emptively to prevent morphological relapse, such as by administering further pre-transplant chemotherapy, increasing the intensity of the transplant conditioning or introducing post-transplant therapy (37). However, the optimal means of using MRD to guide treatment decisions has yet to be fully defined.

When treating relapsed AML with curative intent, allo-SCT is generally the most effective therapy. However, compared to transplants in first remission, outcomes are inferior due to an increase in both relapse (40–45%) and non-relapse mortality (25–35%) (35). Breems et al. developed a prognostic index for AML patients at first relapse based on a study of 667 patients to predict clinical outcomes (41). Three risk groups were defined using four clinical parameters: duration of remission, age at relapse, cytogenetics at diagnosis and whether patients had previously received an allo-SCT. Only 37% of patients in the cohort entered a second complete remission and 18% received an allo-SCT after relapse. However, in all risk groups, patients who received allo-SCT had superior outcomes.

## Evidence for a GvL Effect

A range of donor immune cells are likely to contribute to the GvL response, including T cells, NK, and B cells. However, current clinical evidence suggests that T cells exert the most potent and clinically relevant anti-leukemic effect. Donor T cells recognize leukemia through interactions between their T cell receptor and major histocompatibility (MHC) molecules on the surface of AML cells. MHC, which are known as human leukocyte antigens (HLA) in humans, present peptides to T cells. HLA class I molecules are expressed on all nucleated cells and present peptides to CD8^+^ T cells, whereas class II molecules are primarily expressed on specialized antigen presenting cells and present peptides to CD4^+^ T cells. High levels of HLA class II expression is also seen in most cases of AML (42–44).

When hematopoietic allogeneic stem cell transplantation is planned, a donor is sought with HLA alleles matching the recipient. This reduces immune responses from patient-derived cells, which could result in graft rejection, and from donor-derived cells, which result in GvHD. However, even after a HLA-matched transplant, an immune response against leukemia occurs. The importance of this GvL response in disease control in AML is evidenced by several clinical observations:

1. An inverse correlation between GvHD and disease relapse.
2. Increased relapse in patients treated with T cell depleted grafts.
3. The successful use of DLIs post-transplantation to achieve disease control.
4. Cure of patients treated with reduced intensity conditioning (RIC) regimens that permit stable engraftment of donor hematopoietic cells, but are unlikely to directly kill all leukemia cells (45).
5. Immune evasion mechanisms displayed by AML at post-transplant relapse indicate the presence of immune selection.

### GvHD and Relapse

Many retrospective analyses of patients who received allo-SCT for hematological malignancies have found a significant association between incidence of GvHD and reduced relapse rates (46–49). Following transplantation for AML, Baron et al. found that grade I acute GvHD is associated with lower incidence of relapse (hazard ratio 0.7) and there was a trend toward an OS benefit. However, severe acute GvHD (grade III and IV) was associated with a worse OS due to non-relapse mortality. Similarly, the extent of chronic GvHD correlated with lower relapse rates but increased non-relapse mortality (48). Recipients of identical twin (syngeneic) stem cell transplants have a higher incidence of relapse but a lower treatment-related mortality due to a reduction in GvHD (46).

**Figure 1:**
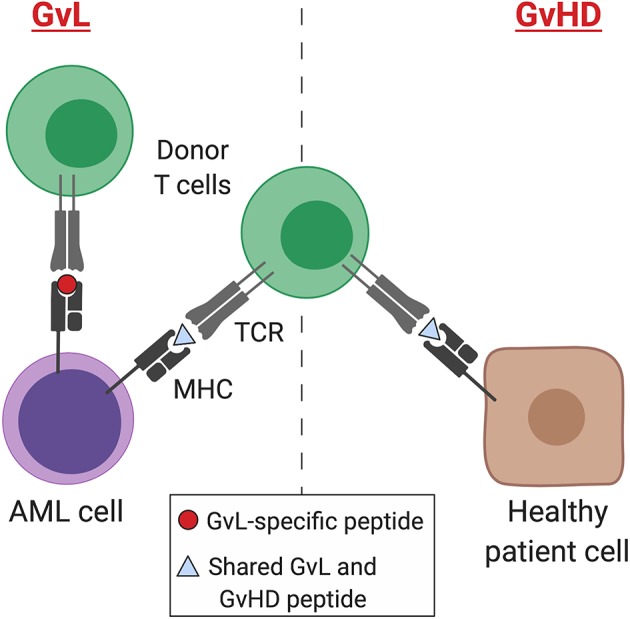
GvL and GvHD T cell responses. Separation of GvL from GvHD T cell responses according to tissue expression of immunogenic MHC-binding peptides. Peptides may result from germline differences between donor and recipient (minor histocompatibility antigens), somatic mutations (neoantigens), or overexpression of non-mutant peptides that are not expressed by healthy tissues (leukemia-associated antigens). AML, acute myeloid leukemia; MHC, major histocompatibility complex; TCR, T cell receptor; GvL, graft-versus-leukemia; GvHD, graft-versus-host disease. Created with BioRender.com.

A retrospective study of 48,111 allo-SCTs performed in adult patients between 1998 and 2007 for a range of hematological malignancies highlighted that GvHD is strongly associated with a GvL effect in CML and ALL, with GvL effect inferred from post-transplant relapse rates. In contrast, there was a weaker correlation between the GvL effect and GvHD in AML and myelodysplastic syndrome (MDS) (45, 50). This suggests that some mechanisms of GvL may be distinct from GvHD and provides hope that these processes may be uncoupled therapeutically (Figure 1).

Studies comparing outcomes of AML patients treated with sibling or matched unrelated donor transplants have yielded conflicting results, with some reporting inferior survival with unrelated donors and others detecting no significant survival difference (51–53). Although a more potent GvL effect may be expected with HLA-matched unrelated vs. related donors, studies have failed to demonstrate a reduction in relapse following unrelated donor transplants (52, 54). It is possible that high resolution HLA typing and advances in supportive care have improved the outcomes of matched unrelated transplants in recent years by reducing transplant mortality (55). However, 7/8 mismatched unrelated donor transplants are associated with increased early mortality, but beyond 6 months survival rates are similar between donor types (56).

The most common source of donor stem cells is peripheral blood, which accounts for 81% allo-SCTs for AML in Europe (1). Donors are treated with recombinant human granulocyte colony stimulating factor (G-CSF) and sometimes with the CXCR4 antagonist plerixafor prior to harvesting by apheresis (57). A bone marrow harvest is performed less commonly, by aspirating marrow from the pelvis, usually while the donor is under general anesthetic. Some studies indicate a survival advantage using peripheral blood stem cell (PBSC) compared with bone marrow for treatment of malignant disease (58, 59), whereas others have shown no impact on OS (60, 61). PBSC transplants have a higher T cell content and have been consistently associated with more rapid neutrophil and platelet engraftment. Lower relapse rates, suggestive of a GvL effect, was observed in a large randomized trial of PBSC vs. bone marrow donation for hematological malignancy (59). However, other studies have shown no effect. Consistent with the higher T cell content of PBSC grafts, a higher incidence of chronic GvHD has been consistently reported across studies and some studies also report an increase in acute GvHD (58–61). Therefore, peripheral blood is more commonly used as the source of stem cells in the treatment of hematological malignancy due to the potential for a more potent GvL effect and the avoidance of general anesthetic risks for the donor. In contrast, bone marrow harvest is generally preferred for the treatment of non-malignant conditions due to the reduced incidence of GvHD and the absence of benefit conferred by the higher number of alloreactive T cells in a PBSC graft (1).

### T Cell Depletion and Relapse

The commonest causes of death after allo-SCT for leukemia are relapse followed by GvHD (62). The role of T cells in GvHD was established in 1968 by van Dicke and colleagues, who used a murine transplantation model to show that mice transplanted with lymphocyte-depleted spleen fractions survived without GvHD, whereas mice receiving lymphocyte-replete fractions all died from GvHD (63). When introduced in human transplants, it was hoped that performing T cell depletion would reduce morbidity and mortality from GvHD and also eliminate the requirement for post-transplant treatment of patients with cyclosporin/methotrexate immunosuppression. Whilst a low incidence of GvHD was confirmed with T cell depleted grafts, there was an increase in graft failure and disease relapse (64).

An early strategy for *ex vivo* T cell depletion of grafts was incubation with Campath-1H (alemtuzumab), the first humanized monoclonal antibody, together with complement from donor serum (Table 2) (65, 66). Although this reduced the incidence of GvHD in patients transplanted for chronic myeloid leukemia (CML), the incidence of relapse approximately doubled (67). Similarly, early experience in AML transplants found an increase in relapse with T cell depletion (46, 68). Marmont et al. studied 1154 AML found a 2.75-fold increased risk of relapse following T cell depletion. An increased incidence of graft failure was observed in both matched related and unrelated donor transplants, suggesting that donor T cells might be required to counterbalance the effect of recipient T cells rejecting the graft (69). These findings suggested that *ex vivo* pan-T cell depletion strategies are not optimal even for unrelated donor transplantation (70). An alternative method of T cell depletion uses CD34^+^ selection of G-CSF-mobilized PBSCs, which results in 4–5 log_10_ reduction in T cells (9). Due to significant reduction in GvHD, this strategy has enabled the transplantation of older patients. Despite concerns about increased relapse due to a reduction in donor T cells available for a GvL effect, this was not seen in a study of AML patients (71).

**Table 2:** Methods of T cell depletion.

**Depletion strategy**	**Method**
***Ex vivo***	
**Positive selection**	CD34 selection
**Negative selection:**	
Pan-T cell	CD3 depletion
	Monoclonal anti-CD52 (Alemtuzumab)
T cell subset	CD8 depletion
	CD3/CD19 depletion
	αβ T cell/CD19 depletion
***In vivo***	
Pre-transplant conditioning	Monoclonal anti-CD52 (Alemtuzumab)
	Polyclonal anti-thymocyte globulin (ATG)
	Atgam® (horse)
	Thymoglobulin (rabbit)
Post-transplant	Cyclophosphamide

*Depletion of T cells may be achieved by manipulating the stem cell graft ex vivo or by administering treatment to the transplant recipient in vivo*.

Other methods of negative selection have been used to balance beneficial effects of donor T cells (a GvL effect and improved engraftment) against GvHD. Treatment of 41 patients with CD8^+^ depleted grafts led to engraftment of all recipients. However, GvHD risk was not reduced, with 61% grade II-IV reported, implying that other immune cell populations are also likely to be important for GvHD such as CD4^+^ T cells or NK cells (72). Combined elimination of T cells and B cells by targeting CD3^+^/CD19^+^ has been used to eliminate both T cells that are responsible for GvHD and B cells that can lead to post-transplant lymphoproliferative disorders. CD3^+^/CD19^+^ depletion was used in a study of 61 recipients of haploidentical transplants who lacked a HLA-matched donor. In this cohort of high-risk patients, 62% had AML and the incidence of grades II–IV acute GvHD and chronic GvHD were 46 and 18%, respectively and incidence of relapse was 31% (73).

The majority (95%) of circulating T cells have T cell receptors that comprise dimers of α and β glycoproteins and are implicated in the adaptive immune response that mediates GvHD and GvL. In contrast, γδ T cells are part of the innate immune system and pre-clinical models have indicated they do not cause GvHD but promote alloengraftment (74). Given the protective role for γδ T cells, there has been interest in specific αβ T cell depletion. Reduction in acute leukemia relapse was observed among recipients of αβ-depleted haploidentical donor grafts compared with pan-CD3 depletion (75). The level of circulating γδ T cells post-transplant correlates significantly with relapse-free survival, suggestive of a GvL effect (76). Furthermore, several subsequent studies have demonstrated anti-tumor activity of γδ T cells against leukemia and other cancers, highlighting their protective role (70, 77, 78).

An alternative approach is to T cell deplete *in vivo* by treating the recipient with either alemtuzumab (79) or anti-thymocyte globulin (ATG) (80) as part of pre-transplant conditioning, or by treating with post-transplant cyclophosphamide (81). Alemtuzumab-containing regimens are consistently associated with a reduction in acute and chronic GvHD (82). Malladi et al. retrospectively studied 88 AML patients treated with HLA-identical sibling transplants, with or without alemtuzumab conditioning. A significant reduction in chronic GvHD was seen and a trend toward increased relapse (35% with alemtuzumab and 19% in the untreated group) (83). Furthermore, due to more pronounced immunosuppression compared with ATG, alemtuzumab is more commonly associated with reactivation of cytomegalovirus (CMV) and Epstein Barr virus (EBV) (82, 84).

High-dose cyclophosphamide administered early post-transplantation depletes alloreactive T cells derived from host and donor. This approach has grown in popularity, particularly in the haploidentical allo-SCT setting, due to its efficacy at reducing GvHD and graft failure (32). Cyclophosphamide selectively targets highly proliferative alloreactive T cells early post-transplant, while sparing the relatively quiescent non-alloreactive T cell and hematopoietic stem cell compartments (27). Ciurea et al. reported a non-randomized comparison of 65 haploidentical transplants: T cell replete bone marrow transplants treated with post-transplant cyclophosphamide vs. T cell depleted PBSC transplants without post-transplant immunosuppression. A significant reduction in chronic GvHD was observed in the T cell replete group (8 vs. 18%) with an associated reduction in non-relapse mortality (16 vs. 42%) and increase in OS at 1-year post-transplant (66 vs. 30%). Improved reconstitution of T cell subsets and a concomitant reduction in infectious complications was also observed in the T cell replete group (85). Following its success in haploidentical allo-SCT, post-transplant cyclophosphamide has been demonstrated to provide effective GvHD prophylaxis and a good safety profile in the HLA-matched related and unrelated donor settings (86).

T cell depletion has been essential in enabling allo-SCTs to be delivered outside of the HLA-matched sibling setting, where the risk of GvHD would be otherwise high, such as mismatched sibling, unrelated donor and haploidentical settings. A balanced approach to T cell depletion is needed, tailored to the GvHD risk associated with the type of donor, and the post-transplant immunosuppressive therapy used.

### DLIs Reinstate a GvL Effect

Given the crucial role of a donor immune response in preventing relapse, in 1989 prophylactic infusions of donor lymphocytes were trialed immediately after transplant in AML patients in an attempt to establish a GvL response (87). However, acute GvHD and mortality was increased. Subsequent work in the 1990s on CML established that using the original transplant donor and separating DLI from the transplant by at least 2 months produced complete hematological, cytogenetic and molecular remissions, indicating a robust GvL effect, often without accompanying GvHD (88, 89). Of relapses, 67% achieved molecular remission following DLI and this translated into 95% 3-year survival. However, transplantation for CML in the current era has been marginalized by targeted tyrosine kinase inhibitor treatment, which is highly efficacious while sparing patients the toxicity of transplantation.

Patients with AML or MDS have a lower response rate to DLI at 20–40% (90, 91). A large retrospective analysis by EBMT of 399 post-transplant AML relapses, of whom 43% were treated with DLIs, showed a survival advantage at 2 years with DLI treatment (21% with DLIs vs. 9% without) (92). Although a GvL effect is evident from DLIs in AML, efficacy has been limited to patients with favorable cytogenetics or a low disease burden at relapse. AML cells are highly proliferative, which may cause them to rapidly overwhelm the ability of donor T cells to exert immunological control in the presence of abundant disease.

Given that DLIs are most likely to exert a therapeutic effect with lower AML disease burden, there has been significant interest in incorporating prophylactic DLI into transplant regimens. For patients with high risk AML, the FLAMSA regimen incorporated prophylactic DLI. It consists of cytoreduction with fludarabine, cytarabine, and amsocrine followed by either 4 Gy TBI or busulphan conditioning, ATG and cyclophosphamide. Prophylactic DLI was given in a non-randomized study at day +120 in patients who were not receiving immunosuppression who were free from GvHD (93). Long-term follow-up revealed that at 7 years post-transplant, there was a significant survival advantage of DLI, with OS 67% compared with 31% in a non-randomized control group (94). A phase II randomized trial of prophylactic DLI post-transplant in high risk myeloid malignancy is currently underway (PRO-DLI, NCT02856464) (95).

### Cure of Patients Treated With RIC Conditioning

Given that the median age of AML diagnosis is 65–70, delivery of transplants with myeloablative conditioning would be associated with unacceptably toxicity and high transplant-related mortality in the majority of patients (4). The development of RIC protocols has enabled allo-SCT to be delivered to older patients. This is particularly important because higher risk cytogenetic and molecular genetic features are commoner in older people and so this cohort would have a limited prognosis if treated with chemotherapy alone.

Myeloablative conditioning regimens use alkylating agents, with or without TBI, at doses predicted to prevent autologous hematopoietic recovery. In RIC regimens, doses of alkylating agents or TBI are reduced by at least 30% (96). RIC must be sufficiently intense to prevent graft rejection by host hematopoietic cells, but preserve a GvL effect, which is especially important in given that leukemia cells are unlikely to be entirely eliminated by the conditioning. A large retrospective analysis of 1,070 AML and MDS patients who received a RIC transplant in first complete remission demonstrated that this treatment can be safely delivered to older adults. The 2-year OS in AML patients aged 65 years or older was 36%, which was not significantly different to survival of younger adults in a multivariate analysis (97). Overall, compared with myeloablative transplants, most data show increased relapse following RIC conditioning, but this is likely to be offset by a reduction in non-relapse mortality, particularly in older adults (98).

### Immune Evasion Mechanisms at Post-transplant Relapse

**Figure 2:**
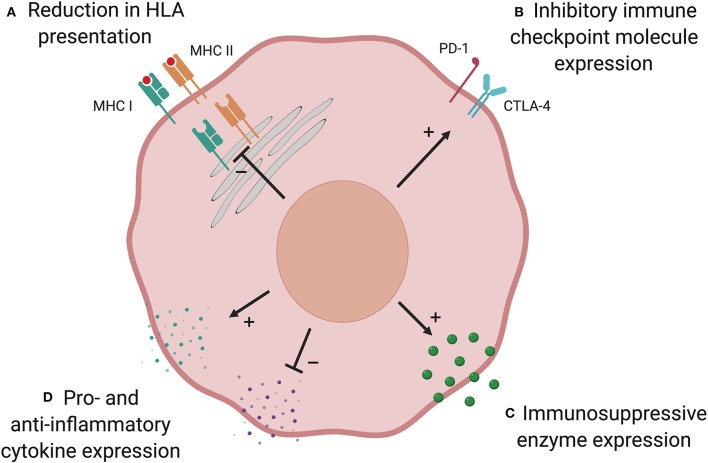
Possible mechanisms of post-transplant immune evasion. Loss of AML immunogenicity under immune selective pressure following allo-SCT leads to immune escape and relapse. There are multiple possible mechanisms: **(A)** Reduction in HLA presentation prevents donor-derived T cells identifying the AML cell. This is commonest in HLA-mismatched transplants and can result from genetic loss of part, or all, of the HLA locus. In other patients, downregulation of HLA expression via defects in transcriptional regulators may play a role. **(B)** Upregulation of immune checkpoint inhibitory molecules has been shown to suppress T cell responses at relapse in a subset of patients. Expression of anti-inflammatory enzymes **(C)** and cytokines, and suppression of pro-inflammatory cytokines **(D)**, have an immunosuppressive effect in AML but their role is yet to be established in the allo-SCT setting. Created with BioRender.com.

T cells play a major role in shaping the immunogenicity of cancers, which can lead to tumor cells altering their phenotype such that they are no longer controlled by the immune system, in a process known as “immunoediting” (99). Loss of AML immunogenicity under donor immune selective pressure has been shown to lead to immune escape and relapse. There are multiple possible mechanisms that may be used by AML to evade the GvL response (Figure 2).

#### Reduction in HLA Presentation

Following haploidentical transplantation for AML or MDS, disease from 5 of 17 patients who relapsed had genomic loss of the mismatched HLA haplotype due to acquired uniparental disomy of chromosome 6p (100). This suggests that in these patients, major HLA mismatches were the critical targets of the GvL response and loss of mismatched HLA as a single large-scale genetic event was sufficient to evade immune detection. Genomic HLA loss has also been described following HLA-matched allo-SCT for AML (101, 102). In a recent study of relapsed AML in 12 recipients of matched unrelated donor transplants, 2 patients were found to have focal deletions spanning HLA class I alleles. Given that HLA alleles were identical in patient and donor, it is likely that the pivotal GvL targets were peptide(s) presented by HLA. In this setting, loss of the HLA locus prevented these key peptides being presented to donor-derived T cells (102).

Recent studies have shown that patients with relapsed AML post-allo-SCT have dysregulation of pathways that influence immune function, including downregulation of the expression of HLA class II genes and associated transcriptional regulators (43, 44). Transcriptional downregulation of genes involved in antigen processing and HLA presentation, such as *IFI30, HLA-DMA, HLA-DMB*, and *CD74*, were observed at relapse in patients with reduced HLA class II expression. In contrast with genetic loss of HLA, reduction in HLA class II expression did not correlate with the number of donor-recipient HLA mismatches and also occurred after HLA-matched transplants, where it may favor immune evasion by substantially narrowing the repertoire of antigens presented to donor-derived T cells. Given that immune evasion mechanisms have involved genomic loss or transcriptional downregulation of both HLA class I and II molecules, roles for CD4^+^ as well as CD8^+^ T cells in GvL responses are likely.

#### Immune Checkpoint Molecule Expression

The balance of co-stimulatory and co-inhibitory signals conveyed to T cells influences whether the cell becomes activated when its T cell receptor binds its cognate HLA-peptide complex. Upregulation of co-inhibitory signaling has been described in many cancers, including AML, and can result in a dysfunctional “exhausted” T cell state (103, 104). In AML patients, CD8^+^ cytotoxic T-cells expressing co-inhibitory receptors are functionally impaired and predict AML relapse (105). Toffalori et al. demonstrated an increase in co-inhibitory ligand expression (PD-L1 and B7-H3) at post-transplant relapse compared with paired diagnostic samples, with a concomitant increase in the corresponding receptors on T cells (44). Increased expression of co-inhibitory ligands and their receptors was mutually exclusive with downregulation of HLA class II, indicating distinct mechanisms of post-transplant immune evasion. Together, these modalities accounted for over two-thirds of relapses.

#### Immunosuppressive Enzymes

Expression of metabolic enzymes by AML, such as arginase II (106) and indoleamine 2,3-dioxygenase 1 (IDO1) (107), has been shown to provide an immunosuppressive environment. AML blasts express and secrete arginase II, resulting in significantly elevated plasma levels and enhanced arginine metabolism compared with healthy controls. This is associated with impaired T cell proliferation and an immunosuppressive M2-like monocyte phenotype (106). IDO1 is an interferon (IFN)-γ-inducible enzyme that metabolizes tryptophan, leading to kynurenine production. Kynurenine inhibits effector T cell function and promotes the differentiation of immunosuppressive regulatory T cells (Tregs). Correspondingly, IDO1 expression by pediatric AML cells was associated with significantly worse 8-year event free survival (16.4%) than non-expressing AMLs (48.0%) (107). Although these mechanisms have not been demonstrated in the allo-SCT setting, they could play a role in suppressing alloimmune GvL responses (108).

#### Alteration of Pro-and Anti-inflammatory Cytokines

A potential method of immune evasion following allo-SCT is the modulation of pro- and anti-inflammatory cytokines. Suppression of pro- and elevation in anti-inflammatory cytokines are predicted to dampen effective GvL responses, though this has not been proven. AML cells express anti-inflammatory cytokines interleukin-4 (IL-4) and IL-10, both of which are known to reduce HLA class II expression (108, 109). This mechanism has been shown to promote immune evasion in a mouse model of chronic lymphocytic leukemia (CLL) (110).

Conversely, pro-inflammatory cytokines could promote GvL responses and therefore suppression of these cytokines might be used by leukemia cells to evade alloreactivity. IL-15 is a pro-inflammatory cytokine expressed by multiple cell types, including dendritic cells and myeloid progenitors (111). Reduced plasma levels early post-transplant is significantly associated with relapse, indicating reduced immune control of disease (112). Furthermore, treatment of AML harboring the internal tandem duplication in the gene encoding Fms-related tyrosine kinase 3 (FLT3-ITD) with the tyrosine kinase inhibitor, sorafenib, increases IL-15 expression in human AML. In a mouse model of AML with FLT3-ITD, sorafenib treatment promotes a GvL effect through IL-15 production (113). A recent phase 1 trial evaluated the IL-15 superagonist complex, ALT-803, in patients with hematological malignancies who relapsed after allo-SCT. Responses were observed in 19% of evaluable patients with expansion and activation CD8^+^ T cells and NK cells, without an increase in Tregs (114). Further study is required to demonstrate whether therapeutic modulation of cytokines can enhance clinically relevant GvL effects and reduce relapse.

## Candidate GvL Antigens

Genetic differences between donor and recipient are crucial for a GvL effect, as is evident from the increased relapse rate seen in syngeneic twin transplants (46). When allo-SCT is planned, a donor is sought with human lymphocyte antigens, HLA-A, -B, -C, -DRB1, and -DQB1 alleles matching the recipient to reduce the risk of GvHD. Often HLA-DPB1 is not taken into account when selecting donors, as OS is unaffected by mismatching HLA-DPB1 alleles. However, mismatches have been associated with an increased risk of GvHD and reduction in relapse, consistent with a GvL effect, suggesting that mismatched HLA-DP may represent important antigens in otherwise HLA-matched allo-SCTs (115, 116).

However, even in the setting of a fully HLA-matched transplant, immune reactivity against malignant cells provides a major contribution to disease control. Donor T cells recognize host antigens through interactions between their T cell receptor and peptide bound to a HLA molecule. A particular HLA allele, together with the peptide being presented, form a complex that is recognized by T cell receptors. T cells play a key role in GvL, even in the HLA-matched setting, which provides evidence that HLA-presented peptides are capable of stimulating an anti-leukemic response (6, 117).

HLA-presented peptides responsible for eliciting a GvL response are likely to result from mismatched coding germline variants between patient and donor that are expressed in leukemia cells (11). These polymorphic peptides are known as minor histocompatibility antigens (miHAs). Most result from single nucleotide variants (SNVs) but insertions/deletions (indels) can also produce miHAs. In addition to germline variants, somatically acquired genetic variants may result in antigenic peptides, which are known as neoantigens. These are formed from SNVs, indels or chromosomal translocations acquired during leukemogenesis. In order for miHAs or neoantigens to result from germline or somatic genetic variants, they must result in an amino acid change (non-synonymous variants), be expressed in AML cells and presented by HLA molecules. Another category of candidate GvL antigens is leukemia associated antigens, which are derived from proteins overexpressed in leukemic cells that are not expressed in healthy cells (leukemia-associated antigens) (90).

### Minor Histocompatibility Antigens

Compared to neoantigens, there are a large number of mismatched germline variants between patient and donor, which suggests that miHAs might be more important in the GvL response due to the number of potential antigens presented to the donor immune system. From whole exome sequencing of nine patient-donor pairs, an average of 6,445 non-synonymous SNVs were found to be mismatched in an allo-SCT, which provides a large reservoir of potential miHAs (118). Genome-wide SNP array data from donors and their stem cell transplant recipients has demonstrated that the average mismatching for coding SNVs is 9.4% for HLA-identical sibling donors, increasing to 17.3% for HLA-identical unrelated donors (119). Not all non-synonymous mismatched variants will produce immunogenic peptides. They must be expressed, processed in the endoplasmic reticulum and bind HLA with high affinity. Approximately 1 in 200 of these non-synonymous SNPs is predicted to be expressed and capable of generating peptides that can be presented by HLA molecules (120). After presentation, in order to generate an immune response, the HLA-peptide complex must bind a T cell receptor, whose activation is also influenced by the balance of co-stimulatory and co-inhibitory signals.

It is not known whether the number of miHAs triggering a GvL response in a given transplant is very large or whether a small number of antigens play a dominant role. miHAs that have a broad expression across tissues may result in GvHD, whereas hematopoietic-specific miHAs might result in a more restricted GvL response.

In early studies, miHA-specific cytotoxic T cells were identified from post-transplant samples by demonstrating selective cytotoxicity against host but not donor cells (121). In this way, HA-1 to −5 antigens were described and were found to be recognized by T cells in a HLA-restricted manner (122). To identify the HLA-A^*^02:01-restricted HA-2 epitope, HLA-bound peptides were eluted by acid treatment and fractionated by reversed-phase high-performance liquid chromatography (HPLC). Individual peptide fractions were tested for their ability to sensitize HLA-A^*^02:01 cells to lysis by an antigen-specific cytotoxic T cell clone. Tandem mass spectrometry of the sensitizing peptide fraction was then used to identify the amino acid sequence of the HA-2 antigen, which derived from a class I myosin protein (123).

Once it was appreciated that miHAs occurred as a result of mismatched germline genetic variants, more efficient methods were used to identify miHAs using genetic linkage analysis and genomewide association studies (GWAS) (124). In most studies, miHA phenotypes were defined by assessing reactivity of miHA-specific T cell clones to EBV-transformed B cells derived from individual patients (125). Phenotypes were then correlated with SNPs and microsatellite markers throughout the genome, to identify the genetic basis of miHAs (124). This approach was successfully employed to identify the miHAs AAC-1 and ACC-2 resulting from SNPs in the *BCL2A1* gene (126), and LRH-1, from a single base deletion in *P2X5* leading to a frameshift (127).

Clinical data correlating individual miHA mismatches with relapse following allo-SCT are limited, as most studies assessing outcomes have focused on GvHD risk rather than anti-tumor effects (124). However, a large cohort study of 849 patients treated with allo-SCT examined 10 previously described autosomal miHAs and correlated patient-donor mismatches with clinical outcomes (128). They found that mismatched miHAs was associated with higher relapse-free survival only in patients with GvHD, but other studies failed to demonstrate this association, perhaps due to small sample sizes (124). A study of three HA-1- and/or HA-2-positive patients with CML or myeloma demonstrated that expansion of miHA-specific T cells after DLI treatment from their miHA-negative donor coincides with clinical disease responses, suggestive of a GvL effect (129).

Donor/recipient gender mismatching is associated with reduced survival, which supports a role for immune responses against antigens encoded on the Y chromosome. Stern et al. found that male recipients of female hematopoietic stem cell grafts were more likely to experience severe acute GvHD than those receiving male grafts. In contrast, female recipients of male grafts had increased risk of graft rejection and worse survival than gender-matched recipients, likely as a result of immune reactivity in the host-versus-graft direction against Y-encoded proteins (130). Evidence for a GvL effect mediated by reactivity against Y-encoded antigens was provided by a study of 3238 HLA-matched sibling allo-SCT recipients, which found that male recipients of female grafts had a lower risk of relapse of any other sex combination. Female/female and male/male transplants had statistically significant hazard ratios (HR) for relapse of 1.26 and 1.38, respectively, and female recipients of male grafts had a non-significant HR of 1.21 (131). Based on *in vitro* T cell activation assays using samples from sex mismatched allo-SCTs, Y-encoded miHAs have been identified from several genes, including *DFFRY, JARID1D, DDX3Y, PCDH11Y*, and *UTY* (132, 133).

Antibodies against Y chromosome miHAs in female-to-male gender-mismatched transplants 3 months post-transplant have been shown to predict chronic GvHD (134). This highlights the possibility that B cell responses may be important in GvHD/GvL responses and also demonstrates that antibodies against miHAs could represent biomarkers for these processes.

### Neoantigens

In solid tumors with a high mutational burden, such as melanoma and lung cancer, tumor-specific somatic mutations have been shown to result in large numbers of predicted neoantigens (99). In patients with non-small cell lung cancer, higher numbers of non-synonymous coding mutations were associated with improved response to the programmed cell death-1 (PD-1) inhibitor, pembrolizumab (135). The median number of non-synonymous somatic coding mutations in this lung cancer cohort was 200, most of which are predicted to be non-pathogenic “passenger” mutations. In contrast, AML has amongst the lowest mutation burden of any adult cancer, with an average of 13 mutations in genes and 5 in recurrently mutated genes in AML (136). Therefore, in AML the likelihood of mutations leading to neoantigens is relatively low.

Studies to identify neoantigens have used either whole exome or RNA sequencing data from tumor cells to identify non-synonymous mutations (135, 137–140). Sequencing of a germline sample from the same patient may be used as a control to enable somatically acquired mutations to be distinguished from germline variants. RNA sequencing can be used to filter expressed variants, with the potential to be presented by HLA. Artificial neural networks trained on HLA binding data have been used to predict which variants will bind HLA with high affinity (141, 142). Putative HLA class I-binding neoantigens have been tested for their ability to elicit functional T cell responses using ELISpot assays, intracellular cytokine staining and fluorescent- and barcode-labeled tetramers (143). However, only 4% of tested peptides elicited a T cell response (144). This highlights that additional factors beyond HLA binding affinity play an important role in peptide immunogenicity, which likely include protein degradation, peptide processing, and immune tolerance. If mutant and wildtype peptide sequences are similar, immune tolerance to the wildtype sequence is also likely to result in tolerance to the mutant sequence reducing its immunogenicity. This factor is incorporated into a refinement to HLA class I binding predictions, by adding weighting for differences in binding strength between mutant and wildtype peptide sequences (145). Despite the relatively low validation of T cell responses for individual peptides, van Buuren and colleagues were able to detect neoantigen-specific T cell responses in 6 of 8 melanoma patients studied (146). As discussed above, peptides presented by class II are likely to play a key role in eliciting a GvL response against AML (43, 44). However, *in silico* HLA-binding predictions are less robust for class II than class I, which poses a challenge to using this approach to identifying important neoantigens or miHAs.

Peptide directly bound by MHC on the surface of tumor cells can be assayed by immunoprecipitating the MHC molecules, eluting bound peptides and sequencing them by liquid chromatography-tandem mass spectrometry to identify the amino acid sequence. By integrating this data with genetic variants identified by next-generation sequencing, MHC-presented neoantigens can be identified. Peptide-specific functional T cell responses can be elicited by a greater proportion of the neoantigens identified using this immunopeptidomic approach than using *in silico* HLA-binding predictions (147, 148).

One proposed method of cancer immune evasion is the loss of mutations associated with neoantigens that trigger a T cell response. A study of paired non-small cell lung cancer samples taken before immune checkpoint inhibitor therapy and at post-treatment relapse demonstrated that resistant clones had lost neoantigens present pre-treatment (149). A recent study of AML patients similarly predicted neoantigens in diagnostic and relapse samples. However, they failed to find selection against putative neoantigens post-transplant, suggesting that neoantigens are not being targeted by donor-derived T cells (43). This is likely to be because there is a smaller repertoire of somatic mutations available in AML to generate neoantigens.

Nevertheless, neopeptides resulting from common AML mutations have been shown to elicit T cell responses *in vitro*. Greiner and colleagues leveraged the SYFPEITHI database of known HLA-binding peptides to generate mutant NPM1 peptides and tested their ability to activate T cells from patients and healthy controls using Cr^51^ release and ELISpot assays for IFNγ and granzyme B (150). They identified two immunogenic peptides that activated CD8^+^ T cells from 33 to 44% of AML patients tested. It has similarly been demonstrated that a FLT3 internal tandem duplication (ITD) neopeptide can also induce HLA-restricted autologous CD8^+^ T cell responses *ex vivo* (151).

### Leukemia-Associated Antigens

Leukemia-associated antigens (LAAs) are expressed in leukemia but not in most healthy tissues. Candidate LAAs, such as the cancer-testis family of antigens, may be expressed in immune sanctuary sites such as testes, or in a developmental time-restricted manner (e.g., placenta). Other antigens are not leukemia-specific and are expressed at low levels in normal tissue (e.g., adrenal glands, ovaries, endometrium) but may be overexpressed in leukemia. These antigens include PRAME (preferentially expressed antigen of melanoma), WT1 (Wilms' tumor), hTERT (human telomerase reverse transcriptase), RHAMM (receptor for hyaluronan-mediated motility), G250/CA9 (an LAA derived from carbonic anhydrase), survivin and proteinase-3 (152, 153). Outside the transplant setting, LAA expression has been found to correlate with improved OS in AML. In a microarray analysis of 116 AML samples, high levels of mRNA for at least one of three LAAs, G250/CA9, PRAME, or RHAMM was associated with significantly improved OS. Specific T cell responses were demonstrated against LAAs using Cr^51^ and ELISpot assays in 47–70% cases, suggesting that the presence of these antigens could potentially enable effective immune surveillance and disease control.

Unlike miHAs and neoantigens, LAAs are proteins that could be expressed by the transplant donor and therefore central T cell tolerance may prevent these proteins from generating a GvL response.

## NK Cells and the GvL Effect

In addition to the established role of T cells in the GvL response, correlative evidence suggests that natural killer (NK) cells may also play a role as effector cells and the relative contributions from T and NK cells in a given transplant may be influenced by multiple factors, including mode of T cell depletion, conditioning regimen and post-transplant immunosuppression (154). NK cells are lymphocytes that form part of the innate immune system and are the earliest lymphocytes that recover after hematopoietic stem cell transplantation. Therefore, they are available to exert protective anti-microbial and GvL effects before T cell reconstitution. Indeed, high levels of donor NK cell chimerism early post-transplant (day +14) have been associated with a lower rate of relapse (155).

NK cell effector function is regulated by the balance of signaling from activating and inhibitory surface receptors (156). NK cells were initially functionally characterized by their ability to kill tumor cell lines that lack expression of MHC class I molecules, which led Ljunggren and Karre to propose the “missing self” hypothesis, whereby NK cells kill targets due to the lack of MHC class I presentation of self-peptides (157). This recognition occurs from the interaction between inhibitory killer-cell immunoglobulin-like receptors (KIRs) on NK cells and MHC class I on target cells. In addition to inhibitory KIR receptors, NK cells express a variety of additional inhibitory (e.g., CD94-NKG2A) and activating receptors (e.g., activating KIRs, CD94-NKG2C). When an NK cell encounters a target cell, the balance of signals from these receptors determines whether the NK cell will exert a cytotoxic effect.

Downregulation of class I molecules is an important mechanism for evading T cell immune surveillance that has been described in several tumors (158, 159). However, this mechanism can lead to NK cell recognition and activation of cytotoxicity. A counter-mechanism employed by some tumors is upregulation of the non-canonical HLA-E that bind CD94-NKG2 receptors on NK cells and prevent tumor killing (160).

NK cell alloreactivity was demonstrated by Ruggeri et al. (161), who studied 57 transplanted AML patients and showed that KIR ligand incompatibility in the graft-vs.-host direction predicted a reduction in relapse and improved survival. KIR genes are polymorphic and genotypes have been linked to a GvL effect in AML. There are two kinds of haplotype, A and B, which are distinguished by the composition of genes for activating and inhibitory receptors. Type B haplotypes contain more activating genes and correspondingly have been linked to stronger cytotoxic reactions against both virus-infected and malignant cells (45). Significant associations between donors with a type B KIR haplotype and reduced AML relapse post-transplant have been shown, which suggests that a clinically relevant GvL effect might be mediated by NK cells (162).

## Improving Transplant Outcomes in AML

**Figure 3:**
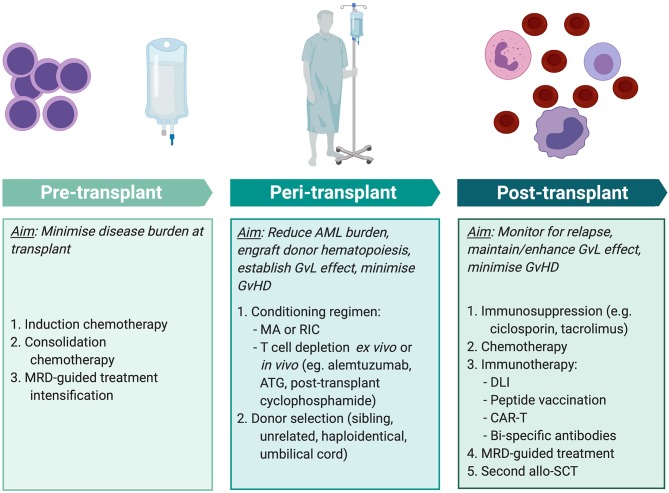
Methods of improving transplant outcomes in AML. Transplant outcomes for AML patients can be improved by optimizing pre-, peri-, and post-transplant factors for individual patients. MRD-guided treatment and some immunotherapy modalities (peptide vaccination, CAR-T, and bi-specific antibody therapy) are not currently standard practice but are areas of exploration. MRD, minimal residual disease; AML, acute myeloid leukemia; MA, myeloablative conditioning; RIC, reduced intensity conditioning; ATG, anti-thymocyte globulin; GvL, graft-vs.-leukemia; GvHD, graft-vs.-host disease; DLI, donor lymphocyte infusion; CAR-T, chimeric antigen receptor T cell therapy; allo-SCT, allogeneic stem cell transplant. Created with BioRender.com.

To maximize the anti-leukemic effect of allo-SCT, multiple factors must be tailored to the individual patient at different stages of treatment (Figure 3). Positive MRD prior to transplant has consistently been associated with inferior outcomes across multiple studies involving myeloablative and RIC protocols (163) and using sibling, unrelated, and haploidentical donors (164, 165). Therefore, the key aim of pre-transplant treatment is to achieve as deep a remission as possible. As discussed above, there is substantial risk of morbidity and mortality from the toxicity of transplant conditioning and from the immunological effects of GvHD. Therefore, when selecting the conditioning regimen and stem cell donor, these risks are balanced against the reduction in relapse that is achieved from intensive conditioning and a GvL effect.

Identification of GvL antigens is essential to understand *in vivo* alloimmune anti-leukemic responses. Elucidating key miHAs may enable improved donor selection such that a genetic mismatch between patient and donor can produce a miHA that is predicted to elicit a T cell response. Whether a mismatch is also likely to result in GvHD may be guided by tissue-specific gene expression data, thus enabling enhancement of GvL in the absence GvHD. It is not known whether GvL responses in individual patients are driven by a small number of dominant T cell clones or if a large T cell repertoire contributes to these responses. Identification of GvL antigens would also enable antigen-specific T cell responses to be tracked in post-transplant samples, allowing us to better understand the key antigens driving responses in individual patients.

Despite an effective GvL response, AML may relapse post-transplant due to the rapid proliferation overwhelming the protective GvL response. MRD assessment post-transplant may predict impending morphological relapse and although not yet established practice, this may enable treatment to be initiated when the disease burden is relatively low and easier to control. This may entail either:

1. Administering post-transplant anti-AML therapy that slows the pace of AML proliferation, to permit an effective donor GvL effect to take hold.
2. Using immunotherapy to enhance the alloreactive effect. This may be targeted to known antigens or employ non-specific immune activation, such as by DLI or immune checkpoint blockade.

Traditionally, post-transplant AML relapses have been treated with chemotherapy, but response rates to treatment are modest and remissions are usually short-lived. Amongst patients who achieve a complete remission with chemotherapy, consolidation treatment with donor cellular therapy, comprising either DLI or a second allo-SCT in selected patients, significantly improves 2-year OS (55% in patients who received cellular therapy vs. 20% in those who did not) (166). However, many patients who relapse following allo-SCT are unable to tolerate further intensive chemotherapy. Treatment with the hypomethylating agent, azacitidine, is associated with relatively low toxicity and has demonstrated efficacy in this setting. Craddock et al. studied treatment of 181 patients with relapsed AML and MDS post-transplant and found that 25% responded to azacitidine but concurrent administration of DLI did not improve response rates (167). It has yet to be established whether, in the absence of relapse, post-transplant maintenance chemotherapy has a role in improving outcomes. Treatment with CC-486, an oral formulation of the DNA methyltransferase inhibitor azacytidine, was well tolerated and associated with a 1-year survival of up to 86% in a single-arm, non-randomized study of AML and MDS patients in post-transplant remission (168). A phase III randomized trial, AMADEUS, is currently evaluating whether maintenance therapy improves relapse-free survival (EudraCT 2018-001012-30) (169). Azacitidine increases the expression of epigenetically silenced antigens and has been shown to result in upregulation of the cancer-testis antigen, MAGE (170). Therefore, in addition to a direct effect on AML cell growth, azacitidine may also enhance the T cell-mediated GvL response.

Our deepening understanding of the molecular landscape of AML in recent years has led to the development of targeted therapies. In relapsed FLT3-ITD AML post-transplant, treatment with the tyrosine kinase inhibitor sorafenib has demonstrated efficacy, both alone and when combined with azacitidine or DLI (171, 172). The second-generation tyrosine kinase inhibitors, quizartinib and gilteritinib, exhibit higher specificity for FLT3 and may be more efficacious (173). A randomized phase III trial of single agent quizartinib in relapsed and refractory AML demonstrated higher CR rates (48 vs. 27%) and prolonged OS (hazard ratio 0.76, 95% CI 0.58–0.98) compared with salvage chemotherapy. However, although patients who relapsed after allo-SCT were included in the trial, this sub-group was not reported separately (174). An ongoing randomized, placebo-controlled phase II trial is currently assessing gilteritinib maintenance therapy, starting at 30–90 days following allo-SCT and continuing for 24 months (NCT02997202) (175).

Mutations in isocitrate dehydrogenase (IDH) enzymes are found in 5–15% of patients with AML and inhibitors to both IDH1 (ivosidenib) and IDH2 (enasidenib) mutant enzymes have been approved by the FDA for treatment of relapsed and refractory AML patients. In this group of patients who are challenging to treat, they have achieved complete remission rates of 30.4% [ivosidenib (176)] and 26.1% [enasidenib (177)]. Both drugs are currently being assessed as maintenance therapy following allo-SCT in patients with AML harboring the relevant mutations (NCT03564821, NCT03515512) (178, 179).

Non-targeted augmentation of post-transplant immune responses using DLI is an established therapy that improves outcomes in some AML patients who relapse post-transplant (92). Another method of non-specific immune activation is using antibodies against immune checkpoint inhibitors, such as PD-1 or CTLA-4. Given their success in treating a range of cancers (180–182), they have also been used in recent studies to treat patients with hematological malignancies following stem cell transplantation. Although these might enhance the GvL response and thereby improve disease control, non-specific immune activation leads to substantial morbidity and mortality from GvHD (183). Concern has also been raised about the possibility of increased risk of veno-occlusive disease (VOD) of the liver with immune checkpoint inhibitor therapy. A retrospective study of lymphoma patients treated with PD-1 blockade prior to RIC allo-SCT found an increased rate of severe VOD compared with a retrospective series of RIC transplants (8 vs. 2.1%) (184). Therefore, it is currently unclear whether immune checkpoint inhibitors will form part of the repertoire of post-transplant therapies for AML.

GvL antigens represent attractive targets for immunotherapy. By selecting a target antigen that is either absent or expressed at a low level in normal tissues, GvHD morbidity can be minimized. One method of producing a targeted GvL response is by peptide vaccination after transplantation, which is predicted to lead to the proliferation of antigen-specific T cell clones. These T cells then target leukemia cells that also present the peptide, leading to effective immune control of disease. WT1 is highly expressed highly in many cancers, including most cases of AML (185). A polyvalent vaccine has been used in early phase clinical trials of AML in complete remission outside the transplant setting, with functional T cell responses demonstrated *in vitro* by IFNγ ELISpot and an increase in WT1 tetramer-positive T cells (186, 187). Chapuis et al. targeted WT1 post-transplant using a TCR gene therapy approach. A high affinity WT1 antigen-specific TCR was inserted into donor CD8^+^ T cells, which were infused prophylactically following allo-SCT into 12 patients. No relapses observed after median follow-up of 44 months (188). Alternative modes of immunotherapy that direct T cells against specific antigens could also be employed to enhance the GvL effect, including bi-specific antibodies (189) and chimeric antigen receptor T cell (CAR-T) therapy (190).

## Conclusion

To achieve a cure, most patients with AML require an allo-SCT. Although this is an effective therapy, 32% of patients will relapse and these cases are associated with poor outcomes (166). This highlights the need to improve treatments to reduce relapse whilst avoiding non-relapse mortality, which is largely due to GvHD and infection. It has been almost 40 years since the GvL effect was first identified in retrospective, correlative clinical studies and although it is clear that T cells play a crucial role, it is not known which AML antigens trigger and sustain a protective GvL donor T cell response in the HLA-matched setting (27). It is probable that GvL responses result from the genetic disparity between patient and donor and given the correlation between GvHD and GvL, these processes are likely to have shared mechanisms. De-coupling the processes that drive GvL responses from those that cause GvHD represents a key challenge in transplantation, that might allow us to harness protective anti-tumor responses, whilst avoiding toxicity to normal tissues.

## Author Contributions

CS wrote the first draft of the manuscript. Both CS and PV reviewed and edited the final version.

### Conflict of Interest

The authors declare that the research was conducted in the absence of any commercial or financial relationships that could be construed as a potential conflict of interest.
